# Identification and functional characteristics of CHD1L gene variants implicated in human Müllerian duct anomalies

**DOI:** 10.1186/s40659-024-00550-w

**Published:** 2024-09-28

**Authors:** Shuya Chen, Yali Fan, Yujun Sun, Shenghui Li, Zhi Zheng, Chunfang Chu, Lin Li, Chenghong Yin

**Affiliations:** 1 Central Laboratory, Beijing Obstetrics and Gynecology Hospital, Capital Medical University, Beijing Maternal and Child Health Care Hospital, 17 QiHeLou Street, Dongcheng District, Beijing, 100006 China; 2 Department of Gynecology, Beijing Obstetrics and Gynecology Hospital, Capital Medical University, Beijing Maternal and Child Health Care Hospital, 251 YaoJiaYuan Road, Chaoyang District, Beijing, 100026 China

**Keywords:** Müllerian duct anomaly, CHD1L, Genetic etiology, Whole exome sequencing, Functional assessment

## Abstract

**Background:**

Müllerian duct anomalies (MDAs) are congenital developmental disorders that present as a series of abnormalities within the reproductive tracts of females. Genetic factors are linked to MDAs and recent advancements in whole-exome sequencing (WES) provide innovative perspectives in this field. However, relevant mechanism has only been investigated in a restricted manner without clear elucidation of respective observations.

**Methods:**

Our previous study reported that 2 of 12 patients with MDAs harbored the CHD1L variant c.348-1G>C. Subsequently, an additional 85 MDAs patients were recruited. Variants in CHD1L were screened through the in-house database of WES performed in the cohort and two cases were identified. One presented with partial septate uterus with left renal agenesis and the other with complete septate uterus, duplicated cervices and longitudinal vaginal septum. The pathogenicity of the discovered variants was further assessed by molecular dynamics simulation and various functional assays.

**Results:**

Ultimately, two novel heterozygous CHD1L variants, including a missense variant c.956G>A (p.R319Q) and a nonsense variant c.1831C>T (p.R611*) were observed. The variants were absent in 100 controls. Altogether, the contribution yield of CHD1L to MDAs was calculated as 4.12% (4/97). All three variants were assessed as pathogenic through various functional analysis. The splice-site variant c.348-1G>C resulted in a 11 bp sequence skipping in exon 4 of CHD1L and led to nonsense mediated decay of its transcripts. Unlike WT CHD1L, the truncated R611* protein mislocalized to the cytoplasm, abolish the ability of CHD1L to promote cell migration and failed to interact with PARP1 owing to the loss of macro domain. The R319Q variant exhibited conformational disparities and showed abnormal protein recruitment behavior through laser microirradiation comparing with the WT CHD1L. All these variants impaired the CHD1L function in DNA damage repair, thus participating in MDAs.

**Conclusions:**

The current study not only expands the mutational spectrum of CHD1L in MDAs but determines three variants as pathogenic according to ACMG guidelines with reliable functional evidence. Additionally, the impairment in DNA damage repair is an underlying mechanism involved in MDAs.

**Supplementary Information:**

The online version contains supplementary material available at 10.1186/s40659-024-00550-w.

## Introduction

Müllerian duct anomalies (MDAs) are congenital developmental disorders that present as a series of abnormalities within the reproductive tracts of females and are sometimes accompanied by malformations in the ipsilateral urinary tracts or other systems. The incidence of MDAs is approximately 6.7%, yet it increases to 7.3% in patients experiencing infertility and 16.7% in those with repeated spontaneous miscarriages [1, 2]. MDAs can lead to symptoms such as dysmenorrhea, dyspareunia, and impaired reproductive capacity, negatively impacting both physical and mental well-being.

Owing to their complex developmental process, unclear pathogenesis, and diverse clinical presentations, limited information is known about MDAs, especially their causative factors. Genetic factors are linked to the occurrence and development of MDAs [1, 3]. Nevertheless, investigations into the genetic mechanisms underlying MDAs have progressed slowly, in part because of the complex regulatory networks involved and the limited sample size. Recent advancements in sequencing technology, particularly whole-exome sequencing (WES), have provided innovative perspectives in this area. Numerous genes within pathways related to MDAs have been identified, such as *GREB1L*, *TBX6*, *HNF1B*, *LHX1*, *WNT4*, *GATA3*, *BMP4*, and *PAX8*, whereas few have been validated by functional assays and identified as candidates [1, 4–7]. However, identifying the genes relevant to MDAs remains a challenging task.

The chromodomain helicase/ATPase DNA binding protein 1-like (CHD1L) gene encodes a versatile protein that profoundly impacts various cellular processes, such as chromosome remodeling, cell differentiation and development [8]. CHD1L has been identified as a dominant disease-causing gene of congenital anomalies of the kidney and urinary tract (CAKUT) [9, 10]. During the embryonic phase, both the urinary and genital systems develop from the primordial urogenital ridges. The Wolffian ducts (WDs) not only participate in the formation of the urinary tract but also indispensably impact the normal development of the Müllerian ducts. Hence, dysregulations implicated in urinary malformations are likely to affect Müllerian duct development and lead to MDAs. Our previous study reported a CHD1L splice-site variant in two sporadic patients with Herlyn–Werner–Wunderlich syndrome (HWWS), without functional evidence [6]. Considering the potential significance of CHD1L in developmental disorders, efforts have been made to recruit more MDA cases, among which two novel variants have been identified. The biological relevance of CHD1L gene alternations with MDAs requires further validation.

This study investigated the genetic origin of MDAs. Through WES, novel CHD1L variants were revealed, and the association between CHD1L abnormalities and MDAs was elucidated, thus providing critical insights for clinical practice and future research.

## Materials and methods

### Patients and ethical approval

Our previous study reported that 2 of 12 patients with HWWS harbored the CHD1L variant c.348-1G>C [6]. Therefore, an additional 85 Chinese Han patients affected by MDAs and diagnosed and treated at Beijing Obstetrics and Gynecology Hospital, Capital Medical University, from January 2019 to February 2024 were recruited. One hundred unrelated Chinese women with normal phenotypes confirmed by imaging examination and/or hysteroscopy were enrolled as controls. All individuals had 46,XX karyotypes. Two experienced gynecologists conducted separate evaluations of patients’ profiles for the purpose of the study, and agreements were reached. MDAs were discriminated by classification systems, including VCUAM [11], ESHRE/ESGE [12], and MAC2021 [13]. The clinical profiles of the patients are summarized in Table 1. Their body mass indices were within the normal range, their body hair was typically distributed, and the hormone analysis results remained normal. Fc-K-1 did not show any significant clinical symptoms and was diagnosed as a partial septate uterus with left renal agenesis at a check-up when she was 26 years old. Fc-U-46491 exhibited a complete septate uterus with duplicated cervices and a longitudinal vaginal septum. She underwent full-term delivery through a cesarean section owing to abnormalities in the birth canal, a typical complication associated with MDAs. Both patients denied a family history of urogenital abnormalities.

**Table 1:** The clinical profile of the patients with MDAs harboring CHD1L mutations

Patient	Fc-K-1	Fc-U-46491
Age at diagnosis, years	26	28
Height, m; weight, kg; body mass index, kg/m^2^	1.58; 52; 20.83	1.67; 61; 23.53
Baseline parity	Nullipara	Pluripara (cesarean section)
Diagnosis of MDAs	Partial septate uterus with left renal agenesis	Complete septate uterus with duplicated cervices and longitudinal vaginal septum
Other diagnosis	–	Hysteromyoma; endometrial polyps; hypertension
Symptoms relating to MDAs	–	–
Classification		
VCUAM	V0, C0, U1b, A0, MR	V2b, C1, U1c, A0, M0
ESHRE/ESGE	U2aC0V0	U2bC2V1
ASRM	Septate uterus	Septate uterus; longitudinal vaginal septum

*VCUAM* Vagina Cervix Uterus Adnex-associated Malformation, *ESHRE/ESGE classification* The European Society of Human Reproduction and Embryology and the European Society for Gynaecological Endoscopy, *ASRM* American Society for Reproductive Medicine

The research received approval from the Ethics Committees of Beijing Obstetrics and Gynecology Hospital, Capital Medical University (2018-KY-027-01 and 2021-KY-032-01), following the ethical principles set forth in the Declaration of Helsinki.

### WES, in silico analysis and Sanger sequencing

The genomic DNA from all recruited participants was isolated from peripheral blood using a TIANamp Blood DNA Kit (DP348; TIANGEN, China) according to established protocols. WES was carried out on Illumina NovaSeq 6000 sequencers, generating paired-end reads of 150 bp for each reaction. Following variant calling and annotation, cases with CHD1L variants in line with the specified criteria were included: (1) potentially affected protein sequences; (2) minor allele frequencies less than 0.01 in accordance with the Genome Aggregation Database; and (3) algorithm prediction of deleterious or likely deleterious [14]. More detailed information about the methodology was provided in a previous publication [6].

Sanger sequencing was employed to confirm the identified variants. The primer pairs used to verify the polymerase chain reaction (PCR) products are listed in Supplementary Table 1. Sequencing was performed in an ABI 3730 automated sequencer (Applied Biosystems, Foster City, CA, USA).

### Molecular modeling and simulations of CHD1L proteins

The predicted structures of CHD1L and poly(ADP-ribosyl)ation polymerase 1 (PARP1) were obtained from the AlphaFold Protein Structure Database (AF-Q86WJ1-F1 and AF-P09874-F1, respectively) [15]. The mutant proteins were then generated using PyMOL by introducing corresponding modifications to the remaining sites in the initial framework. Subsequently, molecular dynamics simulations were conducted with Gromacs2022.3 software at a constant temperature of 298 K and an atmospheric pressure of 1 bar for 100 ns [16]. The force field AMBER14SB was used, with water molecules as the solvent (Tip3p water model) [17]. The simulation system utilized the steepest descent method for energy minimization and then proceeded to NVT and NPT equilibrium, with a 2 fs coupling constant and a 100 ps duration. The subsequent simulation lasted for 20 ns, during which 10,000,000 steps were carried out with a step length of 2 fs. The Particle Mesh Ewald method was utilized to handle long-range electrostatic interactions. The cutoff distances for calculating electrostatic and van der Waals interactions were set at 12 Å and 10 Å, respectively. Trajectory analysis was performed using the tool within the software. The execution of molecular docking between the CHD1L and PARP1 proteins was simulated in ZDOCK [18].

### C57BL/6 mice

The C57BL/6 mice were kept in specific-pathogen-free animal facilities, where the temperature was maintained at 22–26 °C, with a 12-h light/dark cycle. The animal protocols were approved by the Institutional Animal Care and Use Committee (IACUC) of Beijing Obstetrics and Gynecology Hospital.

### Plasmid construction and transfection

The overexpression plasmids pcDNA3.1(+)-CMV-human CHD1L-wild-type (WT)-3 × Flag (NM_004284) and pcDNA3.1(+)-CMV-human PARP1-HA (NM_001618) were purchased from YouBio Biology (China). Mutants of CHD1L related to the point of interest were generated from the WT plasmid. The empty vector pcDNA3.1(+) was used as the negative control. The procedures for constructing the plasmids for the minigene assay are detailed below. The pcDNA3.1(+)-GFP-WT/mutant CHD1L and pcDNA3.1(+)-mCherry-PARP1 plasmids were both created by adding N-terminal fluorophore sequences and eliminating C-terminal tagged protein sequences from the parent plasmids. Transient transfection was conducted using jetPRIME® Transfection Reagent (101000046; Polyplus, France) following the manufacturer’s instructions.

### Cell culture and reagents

293FT cells were cultured in Dulbecco’s Modified Eagle’s Medium (DMEM) (CT11995500BT; Gibco, USA) supplemented with 10% fetal bovine serum (FBS) (F8687; Sigma-Aldrich, USA), penicillin‒streptomycin (100×) (15070063; Gibco, USA), GlutaMAX™-I (35050079; Gibco, USA) and MEM NEAA (100×) (11140050; Gibco, USA). HeLa cells were cultivated in DMEM supplemented with 10% FBS and 1% penicillin‒streptomycin. The cells were incubated in a cell culture chamber at 37 °C with 5% CO_2_ in a humidified environment.

### Antibodies

The related primary antibodies included GAPDH antibody (AC033; ABclonal, China), DYKDDDDK tag antibody (66008-4-Ig; ProteinTech, China), HA tag antibody (51064-2-AP; ProteinTech, China), anti-β actin antibody (TA-09; ZSGB-BIO, China), anti-CHD1L antibody (ab197019; Abcam, USA) and human PARP1 antibody (13371-1-AP; ProteinTech, China). The secondary antibodies used were anti-mouse/anti-rabbit IgG (H+L) biotinylated antibody (ZB-2305/ZB-2301; ZSGB-BIO, China) and anti-mouse IgG (H+L) cross-adsorbed secondary antibody Alexa Fluor™ 488 (A-11001; Invitrogen, USA). Antibodies were used with appropriate dilutions conforming to standard protocols.

### Minigene splicing assay

It was hypothesized that the CHD1L variant c.348-1G>C might impact the splicing of exon 4; thus, vectors encompassing the WT or mutant fragments were constructed. PCR was used to amplify the sequences containing exon 3 (107 bp), intron 3 (836 bp), exon 4 (115 bp), intron 4 (603 bp), and exon 5 (32 bp) with the addition of ATG and TGA from the templates of the genomic DNA of Fc-H-5/Fc-H-8 and the control. The product was collected using an Agarose Gel DNA Purification and Recovery Kit (DH101; Biomed, China). A restriction enzyme sequence containing the KpnI (5′) and XhoI (3′) digestion sites flanking the whole sequence was subsequently inserted into the multicloning site of the pcDNA3.1(+) empty vector. Total RNA was isolated from the 293FT cells transfected with these plasmids using a HiPure Total RNA Mini Kit (R4111; Magen, China), and cDNA was synthesized by reverse transcription (RT) using TransScript One-Step gDNA Removal and cDNA Synthesis SuperMix (AT311; TransGen Biotech, China). The cDNA was subsequently amplified by PCR using the primers listed in Supplementary Table 2, which flanked the target minigene. The PCR products were separated by agarose gel electrophoresis and sequenced, and the transcripts of the WT and mutant fragments were identified.

### RNA extraction and RT-qPCR

The methods used for RNA extraction and cDNA synthesis are described above. Quantitative real-time PCR (qPCR) was performed in triplicate with the PerfectStart Green qPCR Super Mix (AQ601; TransGen Biotech, China) on a fluorescence instrument (LightCycler 480 II; Roche, Basel, Switzerland). The relative gene expression levels were normalized to the critical threshold of the housekeeping gene ACTB. The primers used are listed in Supplementary Table 2.

### Western immunoblotting

Total cell lysates from the transfected 293FT cells were prepared in RIPA buffer containing protease and phosphatase inhibitor cocktails (P0013B and P1046; Beyotime Biotechnology, China). The protein concentration was quantified using an Enhanced BCA Protein Assay Kit (P0009; Beyotime Biotechnology, China). Equal amounts of lysates were subjected to 8–12% SDS-PAGE and transferred onto a 0.22 μm polyvinylidene fluoride membranes (ISEQ00010; Merck Millipore, Ireland). The membranes were blocked in 5% nonfat milk in TBST (0.1% Tween-20 in Tris-buffered saline). After being probed with appropriate primary antibodies followed by secondary antibodies, the proteins were visualized using Immobilon Western HRP Substrate Luminol Reagent (WBKLS0500; Millipore, USA) and a ChemiDoc imaging system (Bio-Rad, USA).

### Immunofluorescence microscopy

293FT cells were cultivated and transfected with the corresponding CHD1L constructs at the appropriate density. After 48 h, the cells were centrifuged at 400 rpm to adhere to microscope slides (188105; CITOTEST Scientific, China) using a Shandon Cytospin™ 4 cytocentrifuge (Thermo Scientific, USA). The samples were then fixed with 4% paraformaldehyde (P1110; Solarbio, China), permeabilized with 0.3% Triton X-100 in Tris-buffered saline and blocked with 5% bovine serum albumin buffer. The cells were subsequently stained with the primary anti-FLAG antibody and secondary antibody conjugated with Alexa Fluor® 488. After washing with TBST, a drop of VECTASHIELD® antifade mounting medium with DAPI (H1200; Vector Laboratories, USA) was used to stain the nuclei and mount the cells. The sealed coverslips were visualized with an EVOS imaging system (AMF7000; Invitrogen, USA).

### Scratch-wound assay

HeLa cells were transfected with the corresponding plasmids and incubated to reach a confluence greater than 90%. Next, the medium was removed, and the surface of the inoculated cells was gently scraped with a 10 μl pipette tip. After washing with PBS (P1020; Solarbio, China), DMEM supplemented with 0.5% FBS was added. The scratches were photographed at 0, 12 and 24 h. The area in which the cells migrated during the observation period was measured with ImageJ software. The results are presented as the migration rate (%), which was calculated as the ratio of the area of cell migration at 12 or 24 h and the initial area at 0 h.

### Transwell migration assay

HeLa cells were seeded, transfected with target vectors and cultivated for 48 h. The cells were then collected and resuspended in serum-free DMEM to an appropriate density. The upper and lower wells of the plate (3422; Corning, USA) were filled with cell suspension and 600 μl of complete medium, respectively. After incubation for 24 h, the cells that had not passed through the wells were carefully removed using a cotton swab. The cells in the lower chamber were then fixed with 4% paraformaldehyde for 15 min, stained with 0.1% crystal violet solution (G1064; Solarbio, China) for 15 min, and washed with PBS. The cells were photographed and counted.

### Coimmunoprecipitation (Co-IP) assay

293FT cells were seeded and transfected with empty vector or WT or mutant CHD1L plasmids with or without PARP1 constructs. After incubation, the cells were completely lysed with lysis buffer containing a protease inhibitor cocktail (P2181S; Beyotime, China) and centrifuged at 12,500×*g* for 10 min at 4 °C. Magnetic beads were conjugated with anti-FLAG, anti-HA and anti-PARP1 antibodies according to the instructions. Protein extracts were mixed with mouse IgG magnetic beads, which were used as a negative control, or magnetic beads (P2171 and P2108; Beyotime, China) with target antibodies at an appropriate ratio (25:1). The protein/bead mixtures were then incubated on a rotating wheel overnight at 4 °C. The beads were then rinsed with lysis buffer and resuspended in SDS-PAGE sample loading buffer. After denaturation at 98 °C for 5 min, the immunoprecipitated proteins were eluted and further evaluated by western immunoblotting.

### Laser microirradiation

The experiment was conducted according to the thorough methodology elucidated in the published literature [19]. 293FT cells were cultured and cotransfected with GFP-tagged CHD1L plasmids and mCherry-tagged PARP1 plasmids. After 24 h, the cells were plated onto 35 mm glass-bottom dishes (801001; NEST, China) coated with poly-d-lysine. The culture medium was subsequently removed, and phenol red-free medium containing Hoechst 33342 (C1028; Beyotime, China) used for sensitization was added to the dishes. Laser microirradiation was carried out on an A1 HD25 confocal microscope (Nikon Instruments Inc., Japan) equipped with an incubation chamber for the regulation of humidity and CO_2_ levels with a 37 °C heating stage. A 405 nm laser, with a total power output of 405.7 mW, was used to induce DNA damage.

### Statistics

The statistical analysis was performed in GraphPad Prism v9.4.1, and the details are specified in the relevant sections. A P value less than 0.05 was considered statistically significant. The error bars denote the standard deviation (SD).

## Results

### Mutational spectrum of patients with MDAs

**Fig. 1:**
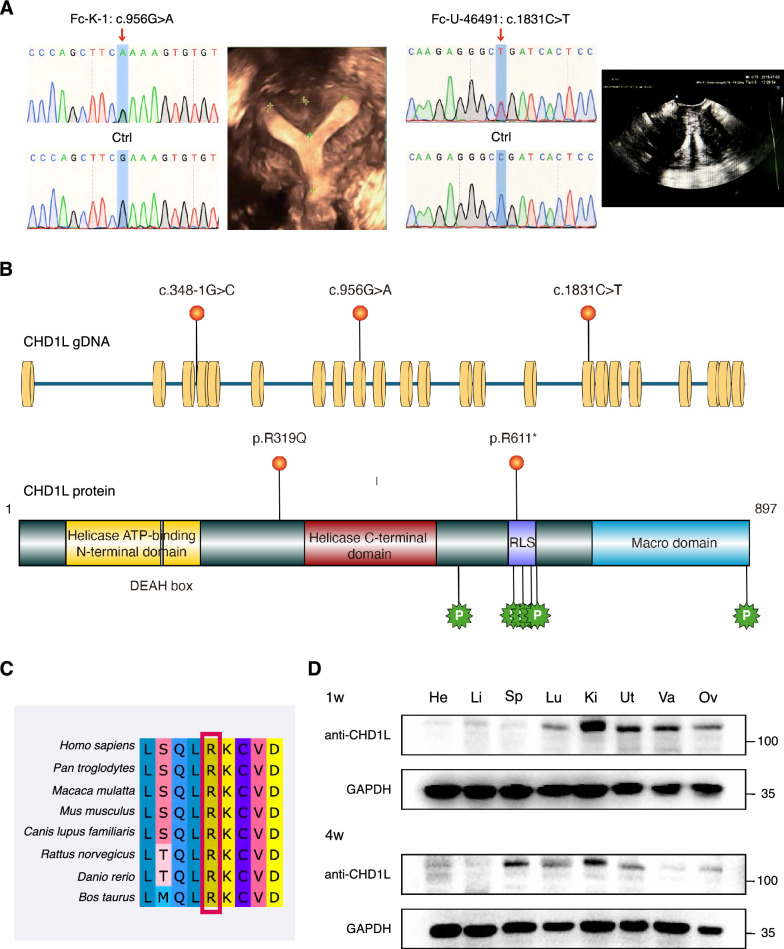
Genetic analysis of CHD1L variants. **A** Sanger sequencing validated in patients Fc-K-1 and Fc-U-46491 and their corresponding ultrasound images. The red arrow indicates the variant site (c.956G>A and c.1831C>T). **B** The scheme for CHD1L gene and protein structures. The human CHD1L gene consists of 23 exons, encoding a protein containing a helicase ATP-binding N-terminal domain, a helicase C-terminal domain, a regulatory linker segment (RLS) and a Macro domain. Variations were indicated with their position. **C** amino acid conservancy in the region of CHD1L surrounding codon 319. **D** Western blot analysis of CHD1L expression in mouse tissues. GAPDH was used as the loading control. *He* heart, *Li* liver, *Sp* spleen, *Lu* lung, *Ki* kidney, *Ut* uterus, *Va* vagina, *Ov* ovary

Despite the previously identified variant c.348-1G>C, according to the WES results, two novel variants of the CHD1L gene (NM_004284) were detected in sporadic patients. Both the missense variant c.956G>A (p.R319Q) and the nonsense variant c.1831C>T (p.R611*) were absent in 100 control patients. The variants were confirmed primarily by Sanger sequencing (Fig. 1A). Located in the regulatory linker segment (RLS), p.R611* is indispensably required to bind the acidic patch of the nucleosome to fully activate the CHD1L remodeler [20]. However, R319Q did not exist in any known functional domain (Fig. 1B). The comprehensive details and computational predictions are summarized in Table 2. All the variants were heterozygous, and no other coherent pathogenic variants related to developmental diseases were identified. The affected amino acid in R319Q was conserved among species (Fig. 1C). Therefore, functional analysis was warranted to further assess the pathogenicity of these strains. CHD1L was highly expressed in the human female genital tracts (Supplementary Figure 1), and we further evaluated its expression in mouse tissues. Notably, CHD1L was expressed significantly but exclusively in the kidney, uterus, and vagina, followed by the ovaries and lungs, in the mice at postnatal Day 7 but was expressed more extensively in the 4-week-old mice (Fig. 1D).

**Table 2:** In silico analysis of the CHD1L variants identified in 2 patients with MDAs

Parameters	Fc-K-1	Fc-U-46491
Mutation type	Missense	Nonsense
Exon	9	16
Variations	c.956 G>A	c.1831 C>T
Amino acids	p.R319Q	p.R611*
Inheritance	Het	Het
GnomAD/ExAC frequency ^a^	0.00003491/0.00006070	0.0003363/0.0004622
Conservation	Conserved	Conserved
In silico predictions		
SIFT	D	NA
PolyPhen-2	PD	NA
MutationTaster	D	D
SNPs&GO	D	NA
FATHMM-MKL	P	UN
AlphaMissense	A	NA
CADD	32.0	40.0
ACMG	P	P

^a ^The Genome Aggregation Database (gnomAD) and the Exome Aggregation Consortium (ExAC) are resources developed by international coalition of investigators, with the goal of aggregating and harmonizing both exome and genome sequencing data from a wide variety of large-scale sequencing projects. In this study, we referred to the allele frequencies in the East Asian (EAS) population. Pathogenicity items: SIFT: D, damaging; PolyPhen-2: PD, probably damaging. MutationTaster: D, disease causing; SNPs&GO: D, disease; FATHMM-MKL: P, pathogenic; UN, uncertain. AlphaMissense: A, ambiguous. NA, not applicable. ACMG items: ACMG, American College of Medical Genetics and Genomics guidelines. P, pathogenic

*Het* heterozygous

### The detected splicing variant results in a frameshift change

**Fig. 2:**
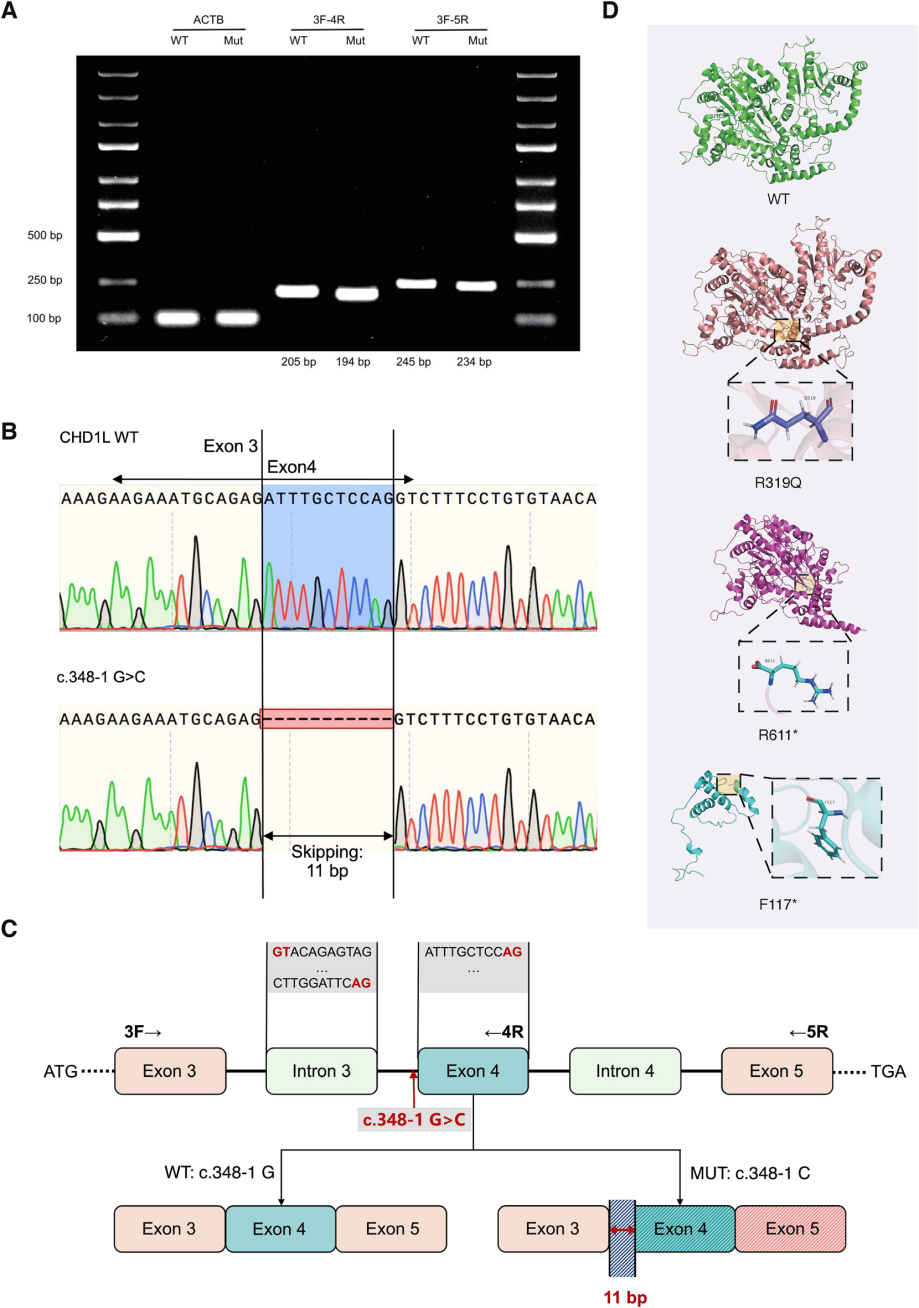
The minigene assay for the splice-site variation and in silico protein models of CHD1L. **A** Agarose gel electrophoresis of the RT-PCR products. Amplified using 3F-4R or 3F-5R primer pairs, the cells transfected with plasmid containing c.348-1G>C variant generated smaller fragment than those with WT plasmids. **B** Sanger sequencing of the RT-PCR products. An 11 bp skipping in the exon 4 of CHD1L caused by the splice-site variation was unveiled. **C** The schematic presentation of the splicing effect of c.348-1G>C. **D** The in silico modeling of the wild-type and mutant CHD1L proteins, with the mutant sites highlighted

The variant c.348-1G>C, located in intron 3, 1 bp upstream of the 5′ end of exon 4 and adjacent to the canonical splice site, was predicted to disrupt the normal splicing of CHD1L. Hence, a minigene assay was adopted. Agarose gel electrophoresis revealed that the number of bands in the mutant plasmid-expressing cell group was slightly but explicitly lower than that in the WT group (Fig. 2A), indicating the occurrence of aberrant splicing events in the mutant group. Sanger sequencing confirmed the elimination of an 11 bp sequence, ATTTGCTCCAG, in the transcript of the mutant group (Fig. 2B). This might be explained by the mechanism by which the c.348-1G>C variation led to the misrecognition of the splice-donor site sequence from 5′-GT…AG-3′, covering the entirety of intron 3, into the initial GT of intron 3 to the AG at the 10th and 11th bases of exon 4. The schematic presentation is illustrated in Fig. 2C. The predicted impact of this frameshift variation was the generation of the CHD1L p.F117Sfs*34 protein. To further investigate its role, a plasmid expressing the p.F117* CHD1L-truncated protein was constructed. The superimposed models of the WT and mutant proteins are shown in Fig. 2D.

### Variants caused loss-of-function effects on the CHD1L gene

**Fig. 3:**
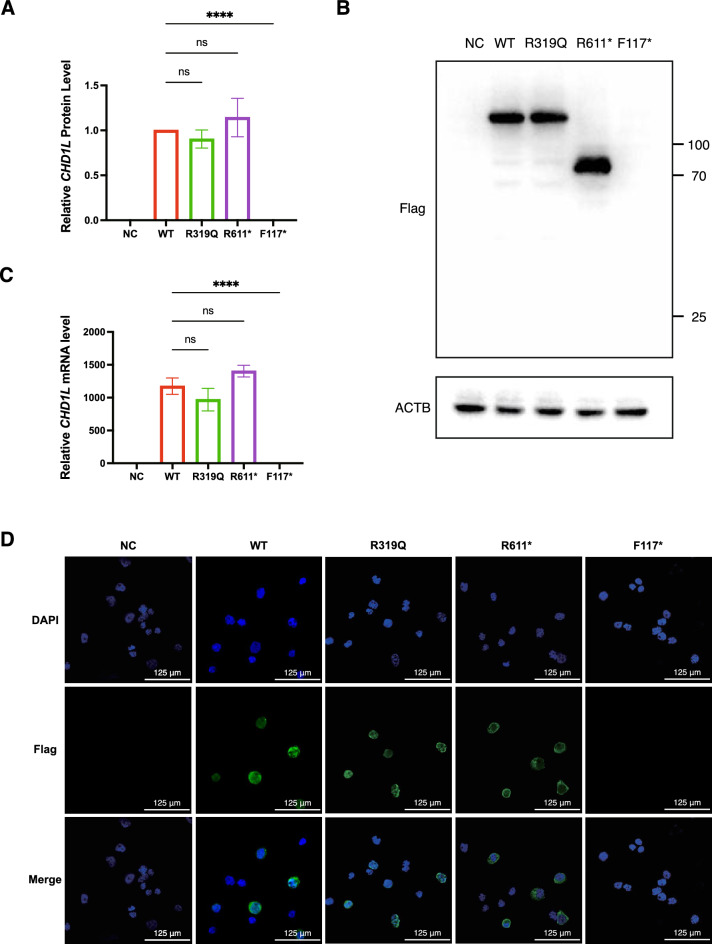
Functional analysis of the pathogenicity of human CHD1L variants carried out in 293FT cells after transfection with WT or mutant CHD1L plasmids. **A** Reverse transcription polymerase chain reaction analyses of CHD1L mRNA level. ACTB was used as the control. **B** Western blot analysis of CHD1L expression. ACTB was used as the loading control. **C** Quantification of CHD1L protein expression. **D** Immunofluorescence staining of WT and mutant CHD1L proteins. The cellular nucleus was visualized using DAPI (blue). The flag-tagged protein was stained in green. In **A** and **C**, data are presented as the mean ± SD of 3 independent experiments. ****P < 0.0001, one-way ANOVA followed by Dunnett’s test

To explore the biological consequences of the CHD1L variants, well-established in vitro functional assays were performed. With respect to mRNA expression and stability, RT-qPCR revealed that both the R319Q and R611* groups maintained levels consistent with that of the WT group. However, the F117* group did not overexpress altered CHD1L transcripts after transfection with corresponding plasmids such as WT but was in accordance with the NC group (Fig. 3A). These findings indicated that the c.348-1G>C variant impaired mRNA stability and most likely triggered the highly conserved RNA surveillance pathway of nonsense-mediated decay. The CHD1L protein expression results were concordant with the qPCR results. The expression levels were similar among the WT, R319Q and R611* groups, whereas no immunoblotting of F117* was detected (Fig. 3B, C). WT CHD1L was localized in the nucleus. Immunofluorescence revealed that the R319Q variant did not interfere with protein localization. However, truncated R611* failed to colocalize with DAPI and appeared in the cytoplasm (Fig. 3D).

### The variants led to conformational changes in the native CHD1L protein

**Fig. 4:**
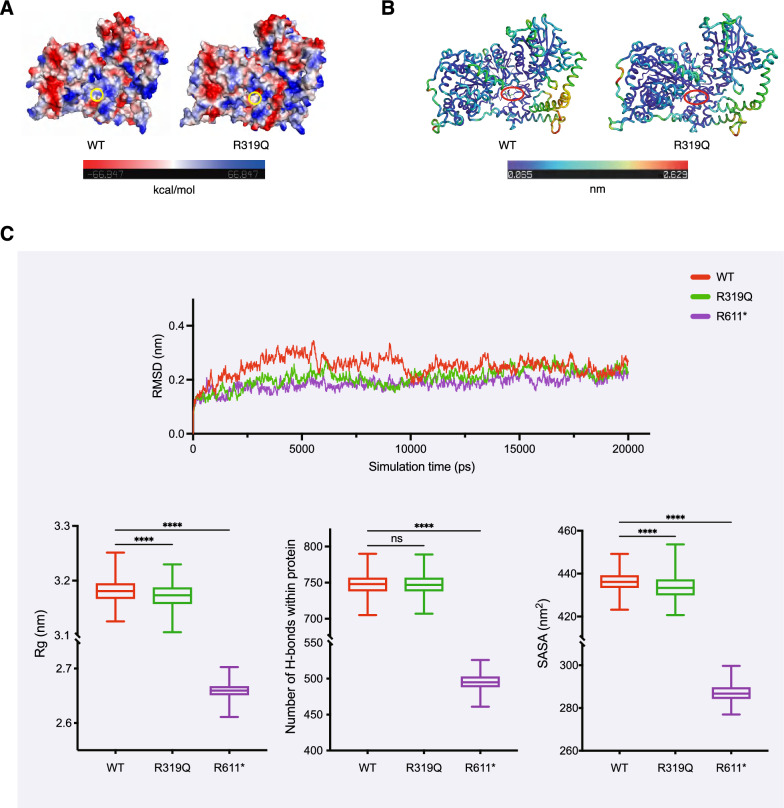
Structural modeling and simulation of CHD1L proteins. **A** The differences of surface electrostatic potential between WT CHD1L and R319Q variant. **B** The assessment of the fluctuations of atoms in WT and R319Q proteins. **C** Molecular dynamics simulations of the WT and mutant CHD1L proteins. Root-mean square deviation (RMSD) of alpha carbons showed WT and mutant CHD1L structures reached stable status during the simulations. The radius of gyration (Rg), the number of H-bonds and solvent-accessible surface area (SASA) at the protein level were depicted using box plots with the minimum and maximum values (whiskers), the upper and lower quartiles, and the median. The length of the box represents the interquartile range. ****P < 0.0001, one-way ANOVA followed by Dunnett’s test

To gain insights into the molecular behavior of the CHD1L variants, in silico molecular simulations were conducted. As illustrated in Fig. 4A, the glutamine substitution of R319 altered the localized electrostatic potential of the CHD1L protein from carrying a positive charge to being electrically neutral. Additionally, the displacement increased the atomic fluctuations around this specific position (Fig. 4B). The alterations could interfere with interactions between proteins, although they were deemed less significant considering the protein’s size. The root-mean-square deviation (RMSD) quantifies the average distance between atoms in a protein and indicates disparities in stability among structures. The RMSD analysis according to time evolution for all Cα atoms, using WT as a reference, indicated that all CHD1L structures achieved stability (Fig. 4C). The radius of gyration (Rg) is utilized to describe the structural compactness of a protein. As depicted in Fig. 4C, the R319Q protein was more compact and stable than the WT protein, with reduced flexibility. The time-dependent hydrogen bond (H-bond) formation assay demonstrated that the number of H-bonds within the proteins decreased drastically in the truncated R611* group but was similar in the WT and R319Q proteins (Fig. 4C). Compared with those of the WT, the solvent-accessible surface area (SASA) values of the R319Q and R611* mutants were lower, suggesting that the surface areas of the R319Q and R611* mutants were less accessible to the solvent molecules (Fig. 4C).

### Variants abolish the ability of CHD1L to promote cell migration

**Fig. 5:**
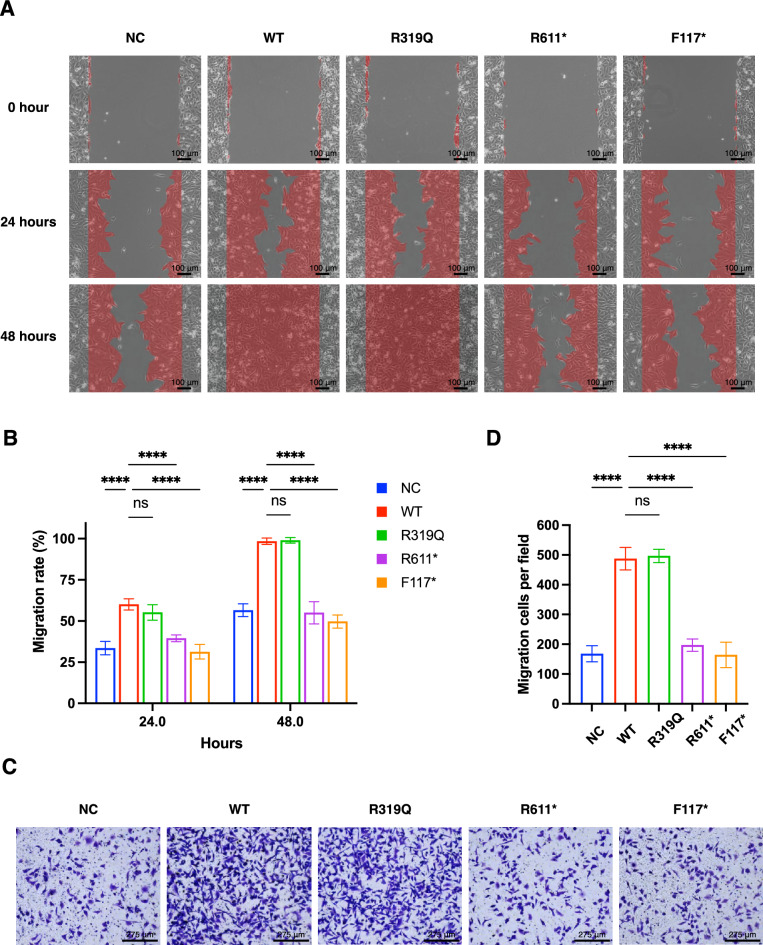
The ability of CHD1L on promoting cell migration. **A** The scratch-wound assay tested in HeLa cells after transiently transfected with WT and mutant CHD1L plasmids. **B** Quantification of cell migration rate at observation points of 24 h and 48 h. **C** Transwell migration assay performed in in HeLa cells after transfected with corresponding plasmids. **D** Quantification of migrated cells per field. In **B** and **D**, data are presented as the mean ± SD of 3 independent experiments. ****P < 0.0001, one-way ANOVA followed by Dunnett’s test

The vital elongation phase of Müllerian duct formation involves the migration of proliferative cells alongside the mesonephros and is governed by sophisticated genetic factors [1]. CHD1L has a strong ability to regulate cell migration through its abnormal expression [8]. These data led to the hypothesis that CHD1L might be involved in Müllerian duct development by regulating cell migration. Herein, the influence of CHD1L variants was analyzed. In the scratch-wound assay, the HeLa cells transfected with the WT or R319Q plasmid presented significantly increased migration rates, both of which were elevated to 100% at 48 h, compared with those of the cells expressing the empty vector (Fig. 5A, B). However, the R611* and F117* groups did not display increased migration abilities. Similarly, in the transwell assay, the number of migrating cells in the WT or R319Q groups was more than twice that in the control group, while the cell counts in the other two groups were similar to those in the control group (Fig. 5C, D).

### Loss of the macro domain of CHD1L abolished its PARP1-driven chromatin remodeling activity

**Fig. 6:**
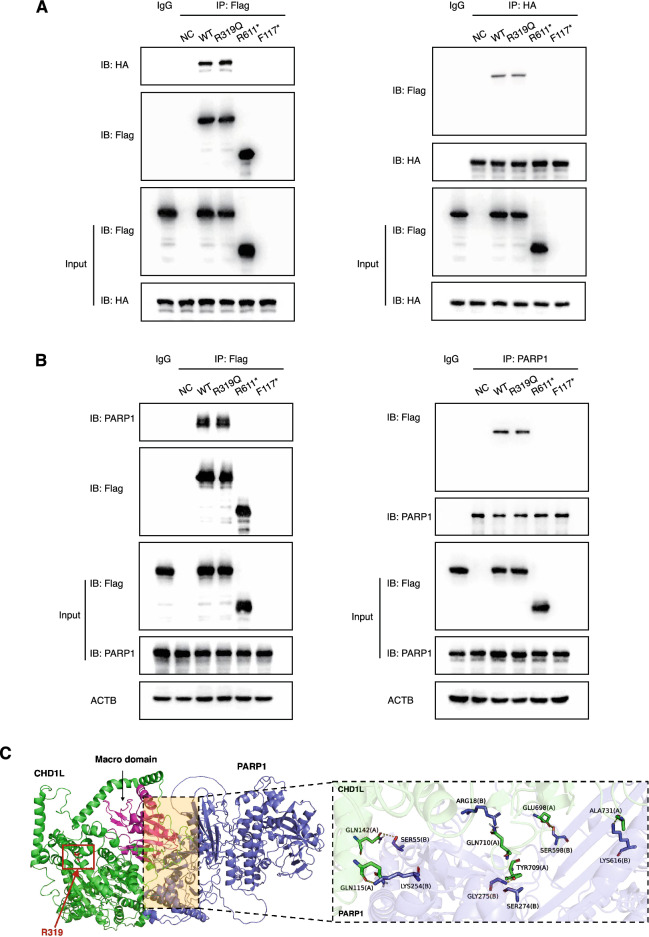
The interaction between WT or mutant CHD1L and PARP1. **A** Co-IP assay performed using cell lysates of 293FT cells co-transfected with exogenous flag-tagged CHD1L and HA-tagged PARP1 plasmids. **B** Co-IP assay between exogenous flag-tagged CHD1L proteins and endogenous PARP1. ACTB was used as loading control. **C** Schematic representation illustrating the molecular docking of Macro domain of human WT CHD1L and PARP1

The macro domain located at the C-terminus of CHD1L is the specific site that interacts with poly(ADP-ribose) (PAR), which is synthesized using NAD^+^ as a substrate regulated by PARP1 in response to DNA strand breaks [21]. This chromatin remodeling function is essential for the ontogenesis of the female reproductive tract. A Co-IP assay was performed to assess the interaction between WT/mutant CHD1L and PARP1. Notably, exogenous HA-tagged PARP1 coimmunoprecipitated with exogenous flag-tagged WT and R319Q mutant proteins but not with R611* proteins whose macro domain and F117* were not degraded (Fig. 6A). Moreover, the interactions between exogenous CHD1L proteins and the endogenous PARP1 protein were also evaluated. Figure 6B shows that the interactions were uninterrupted in the R319Q group, similar to the WT protein, but were abrogated in the R611* and F117* variants. This mechanism might be explained by the molecular docking depicted in Fig. 6C, in which the arginine residue at position 319 of CHD1L, which is located at a distance from its macro domain, did not interact with PARP1 directly.

### The R319Q mutation impaired CHD1L function in DNA damage repair

**Fig. 7:**
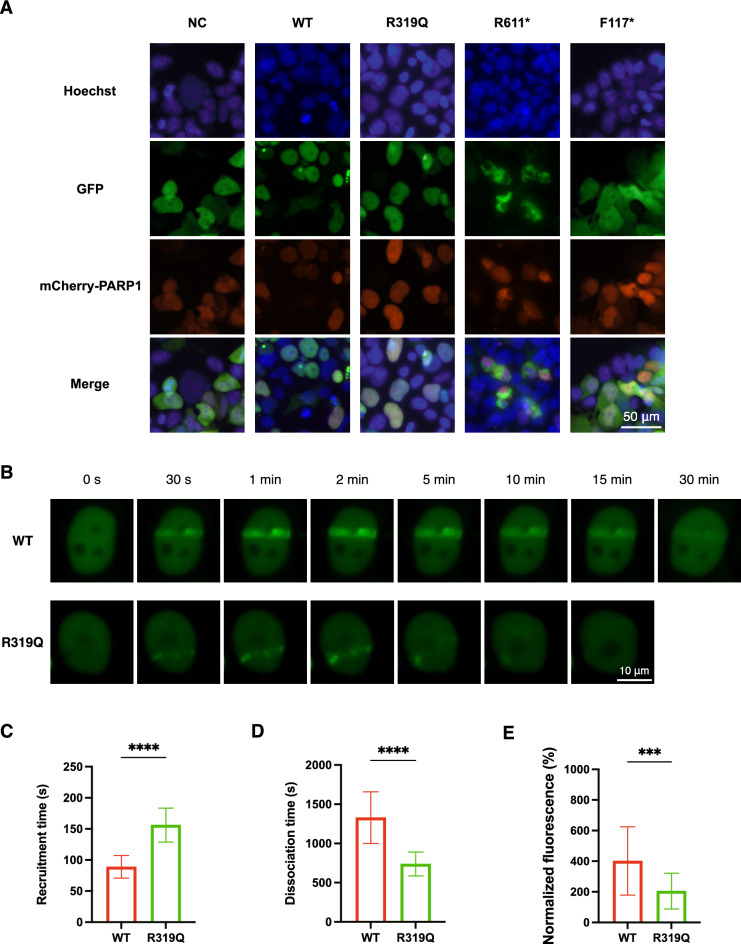
The recruitment of CHD1L to sites of laser-induced DNA damage. **A** The locations of WT or mutant GFP-CHD1L and mCherry-PARP1 in living 293FT cells under fluorescence microscope. The GFP-NC plasmid transfected was used as negative control. The cellular nucleus was stained using Hoechst (blue). **B** Mobilization, association, and dissociation of CHD1L to the specific sites of laser-induced DNA damage. **C**–**E** Quantification of the recruitment time, dissociation time and normalized fluorescence of WT and R319Q CHD1L proteins at DNA damage sites. Plotted data were presented as the mean ± SD of 25 cells. ****P < 0.0001, ***P < 0.001, unpaired, two-tailed Student’s t test

When DNA damage occurs, PARylation via PARP1 mediates the recruitment of CHD1L to a specific site, facilitating chromatin remodeling [20, 22]. The above results revealed complete destruction of the interaction between PARP1 and the R611* or F117* variant, whereas the engagement of the R319Q variant seemed unviolated. To further investigate the behaviors of the mutant proteins involved in the DNA damage repair process, laser microirradiation was performed. First, as depicted in Fig. 7A, the GFP-tagged WT and R319Q CHD1L proteins were correctly colocalized with mCherry-PARP1 in the nuclei of living 293FT cells, whereas R611* was separately positioned with PARP1 and appeared in the cytoplasm. DNA damage was subsequently induced by a 405 nm laser, and WT CHD1L was promptly recruited to specific sites and dissociated gently, whereas the R319Q variant presented delayed translocation and untimely disaggregation (Fig. 7B–D). Furthermore, R319Q failed to reach a postirradiation fluorescence intensity as high as that of the WT protein (Fig. 7E).

Taken together, these data confirmed that all the variants impaired the CHD1L protein. These cases were classified as pathogenic according to ACMG guidelines (Table 2). The contribution yield of CHD1L to MDAs, defined as the percentage of cases, was calculated as 4.12% (4/97).

## Discussion

Both the urinary and genital systems differentiate from the primordial urogenital ridges during embryogenesis. In females, Müllerian ducts remain and form genital tracts through midline fusion, septal resorption, and fusion with the urogenital sinus, whereas the WD ultimately regresses [3]. Nonetheless, the latter plays a vital role in Müllerian duct elongation, as the initiation of this phase is established by the intimate contact of these two ducts [1]. Additionally, the WD might secrete chemoattractants or morphogens to guide cell proliferation caudally [23]. The WD is crucial to the development of the urinary system, and its deficiency can lead to CAKUT [24]. Therefore, the genetic regulatory networks within the intricate and dynamic developmental processes of the urinary and genital systems are interrelated and interact. Any genetic deviation, carrying an inherent risk of urinary abnormalities, may affect Müllerian duct development and ultimately result in MDAs.

The highly conserved gene CHD1L exhibits spatial‒temporal expression patterns in various tissues. In humans, CHD1L is developmentally regulated and expressed at a level that is four times higher in fetal kidneys than that in adult tissues [9], suggesting its crucial role in kidney formation. Hence, its role as a target candidate gene of CAKUT has been investigated [10]. CHD1L is also expressed extensively in female genital tracts [25]. In the present study, signals were detected in the uterus and vagina among the mouse tissue samples. In combination with plausible functional evidence, our data revealed the underlying critical role of CHD1L malfunctions in MDAs.

The CHD1L gene is mapped to chromosome 1q21.1 and encodes a protein comprising 3 major domains. The helicase N-terminal domain and helicase C-terminal domain are crucial units for harnessing energy for DNA events [8]. Located in the C-terminus, the macro domain, which has a high affinity for ADP‒ribose binding, is the region that differentiates CHD1L from other proteins of the CHD family; this region contains a chromatin organization modifier domain that recognizes methylated histone tails [8]. Through cooperation between the macro domain and the PAR moiety of PARP1 at DNA damage sites in the nucleus, CHD1L is activated to drive chromatin relaxation and participate in DNA damage repair [21]. Moreover, chromatin plasticity is critical in organogenesis of the female genital tract, as it orchestrates spatiotemporally developmental events; thus, CHD1L deficits might be implicated in MDAs. Among the CHD1L variations identified in the current study, the c.348-1G>C variant triggered nonsense-mediated decay of its transcript. Compared with the WT protein, the R319Q variant, though localized and expressed normally, caused conformational changes. The R611* variant, located in the RLS region, resulted in the loss of the macro domain, thus hindering its nuclear translocation. Taken together, these variants modify protein structures and have adverse effects on native proteins.

The developmental cascade of Müllerian duct formation can be characterized into initiation, invagination, and elongation. In contrast to the former two phases, which proceed independently, elongation relies on Wolffian signaling. During elongation, proliferative cells migrate along the mesonephros in close proximity to the WD, and this process is subject to labyrinthine and delicate genetic regulation [1]. A panel of genes has been explored, with the exception of CHD1L. WNT9b coordinates elongation through the activation of the canonical WNT pathway [23], whereas a decrease in GATA3 levels results in a halt at later embryonic stages [26]. WNT4 regulates directional cell migration and extension of the Müllerian duct, and ducts fail to form in its absence [27]. Other relevant genes include those attributed to retinoic acid signaling [28, 29]. Abnormal expression of CHD1L affects cell migration [8]. Scratch-wound and transwell assays were conducted, and the results revealed that CHD1L might participate in Müllerian duct development by inducing cell migration, whereas the R611* and F117* variants lack this ability, leading to a truncation of the elongation process.

Once the formation is completed and the WD regresses, the horizontal and caudal regions of the elongated ducts begin to migrate and fuse in the midline to form a tubular structure with a medial septum, after which the septum undergoes resorption, and the ducts eventually merge with the urogenital sinus [3]. Interruptions of these processes can result in uterine anomalies, such as didelphys and septate, as well as aberrant vaginal morphogenesis, such as the longitudinal vaginal septum, which conforms to the manifestations of the patients with CHD1L variants identified in the current study. More robust evidence regarding genes essential for reproductive tract development has been obtained from genetically modified mouse models. For example, in LHFPL2^G102E^ mutant mice, the ducts have defective tip development, affecting fusion, and fail to enter the urogenital sinus. The mutant female mice presented with an abnormal upper longitudinal vaginal septum and lower vaginal agenesis, with an infertility rate of 100% [30]. Similarly, β-catenin has been linked to MDAs because 91% of the homozygous β-catenin^C429S^ mice exhibited vaginal aplasia, and some had the septum concurrently [31]. Notably, a CHD1L knockout mouse model for investigating homologous recombination deficiency cancers has been generated [32]. Although CHD1L loss is compatible with viability and fertility, CHD1L^−^/^−^ mice are born at slightly reduced sub-Mendelian ratios (14% versus 25%) and are smaller in size than WT mice. However, the study did not report the phenotypes of the urogenital tracts of female WT and mutant mice in detail. In clinical practice, concomitant diseases are correlated with the phenotypes and severity of MDAs. Uterine factor infertility is caused by either the absence of a uterus or the presence of a nonfunctional uterus, and among the malformations, the most relevant is Mayer–Rokitansky–Küster–Hauser syndrome [33]. Patients with other abnormalities, such as a septate uterus, are reported to be mostly fertile but may be at risk of preterm delivery, fetal malpresentation, and other complications [34]. In the research conducted by Ludwin et al., there were no significant differences in the prevalence of septate uterus between women with infertility and those without infertility [35]. Furthermore, incomplete penetrance and variable expressivity of MDAs should also be taken into consideration [1].

Chromatin-remodeling enzymes play critical roles in development, and their dysfunction has been shown to be closely related to congenital genital malformations. Aberrations in CHD7, an ATP-dependent eukaryotic enzyme attributed to the CHD family whose structure is homologous to that of CHD1L, have been confirmed to cause CHARGE syndrome, where “G” represents genital and urinary abnormalities [36]. A female patient with a 46,XX karyotype harboring a p.Y835* CHD7 truncation mutation lacked a uterus, vagina, and ovaries [37]. These absences were explained by the disruption of nucleosome remodeling activity caused by variations, interrupting the DNA damage repair process and the additional downstream factors involved in developmental procedures. However, the significance of CHD1L variants has not been explored. In the absence of DNA damage, the interaction of the macro domain of CHD1L with its ATPase remained inactive. CHD1L relieves autoinhibition through the recognition of PAR by the macro domain and subsequently engages with PARP1 to promote DNA repair [22]. This mechanism was substantiated in the current study. By performing a Co-IP assay, R611* and F117* both showed no interaction with PARP1. Although the interaction between the R319Q variant and PARP1 was not undermined, a laser microirradiation assay revealed that the R319Q variant resulted in prolonged recruitment and shortened retention, with lower fluorescence intensity, at the DNA damage site than did the WT. These findings suggested that CHD1L variations resulted in functional deficits in DNA damage repair and interrupted the morphogenesis of the female genital tract.

The main limitation within the present study was the relatively limited sample size of MDAs. Hence, the accumulation of larger patient cohorts, advancements in technologies and genome-wide databases, and the execution of functional analysis are warranted for better interpretation of the pathogenesis and corroboration of the inheritance patterns of MDAs.

## Conclusions

Elucidating the genetic architecture of MDAs is of paramount importance. The current study not only expands the mutational spectrum of CHD1L but also explores the underlying pathogenic mechanisms implicated in MDAs. Taken together, these data provide valuable references for clinical practice and future investigations.

## Supplementary Information

Supplementary Figure 1. Gene expression for CHD1L. The data were obtained from an online database. The red arrow indicates the high expression of CHD1L in the human uterus, cervix, and vagina.

Supplementary Material 2.

## Data Availability

The WES data are available from the corresponding author upon reasonable requests. Other data generated or analyzed during the study are included in this published article and its supplementary files.

## Abbreviations

CAKUT: Congenital anomalies of the kidney and urinary tract
CHD1L: Chromodomain helicase/ATPase DNA binding protein 1-like
Co-IP: Co-immunoprecipitation
FBS: Fetal bovine serum
HWWS: Herlyn–Werner–Wunderlich syndrome
H-bonds: Hydrogen bonds
MDAs: Müllerian duct anomalies
PARP1: Poly(ADP-ribosyl)ation polymerase 1
PCR: Polymerase chain reaction
Rg: Radius of gyration
RLS: Regulatory linker segment
RMSD: Root-mean-square deviation
RT: Reverse transcription
SASA: Solvent-accessible surface area
WD: Wolffian ducts
WES: Whole-exome sequencing
WT: Wild type
