# Supplementary figures and images for: Identification and functional characteristics of CHD1L gene variants implicated in human Müllerian duct anomalies
Source: Biol Res. 2024 Sep 28;57:68. doi: 10.1186/s40659-024-00550-w (PMC11437902; doi:10.1186/s40659-024-00550-w)

Figure S1

A

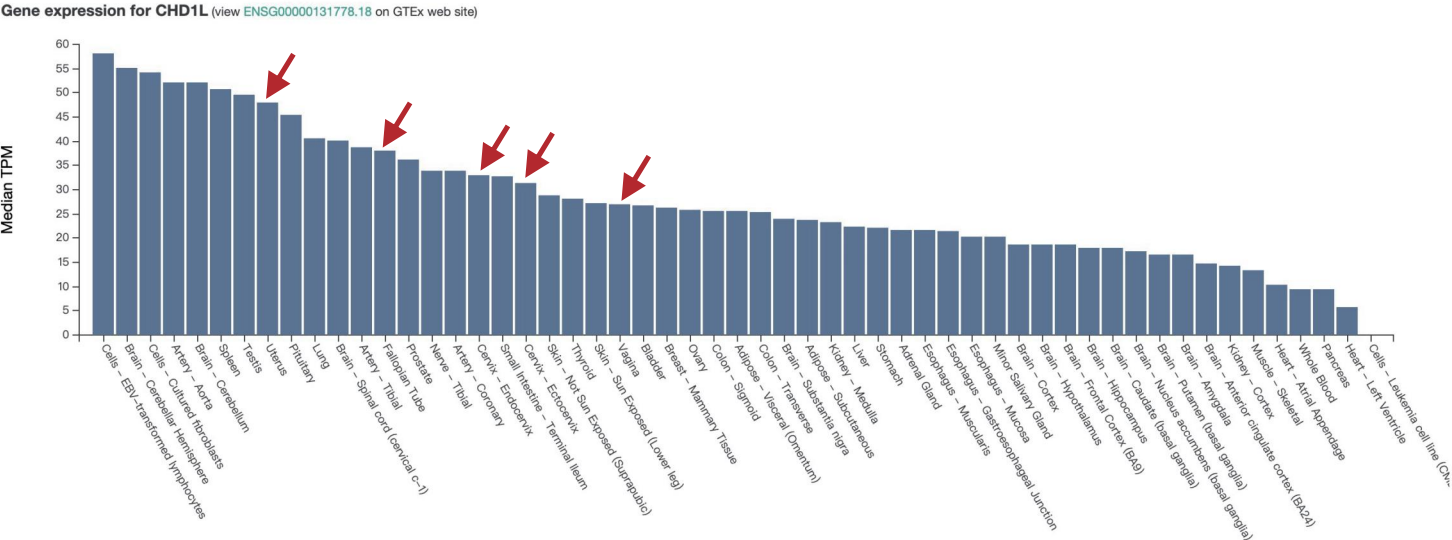

Supplement: Supplementary file 1 — Supplementary Figure 1. Gene expression for CHD1L. The data were obtained from an online database. The red arrow indicates the high expression of CHD1L in the human uterus, cervix, and vagina. [file 40659_2024_550_MOESM1_ESM.pdf]
