# Supplementary material for: Identification and functional characteristics of CHD1L gene variants implicated in human Müllerian duct anomalies
Source: Biol Res. 2024 Sep 28;57:68. doi: 10.1186/s40659-024-00550-w (PMC11437902; doi:10.1186/s40659-024-00550-w)
Supplement: Supplementary file 2 — Supplementary Material 2. [file 40659_2024_550_MOESM2_ESM.docx]

**Supplementary Table 1.** Primers used for Sanger sequencing of patient with CHD1L variants

| Primer name | Sequence (5’→3’) |
| --- | --- |
| p.R319Q-F | GAGGGTCTTTCTTAAAACAAAAT |
| p.R319Q-R | TCGTGGATTTAACAAAAAAAGA |
| p.R611X-F | AAGAGCCCAGTAAGGAAGACAG |
| p.R611X-R | GAGAACAAAACGATGCCAGC |

**Supplementary Table 2.** Primers used in minigene assay and RT-qPCR

| Primer name | Sequence (5’→3’) |
| --- | --- |
| Mini-ATG-F | ATGACTATTGCTCTCTTCATTTATTTGGCAG |
| Mini-TGA-R | TCAATTTTAGAAATGATGCATCTTTCAAGCA |
| Mini-KpnI-F | CCGGGGTACCATGACTATTGCTCTCTTCATTTATTTGGCAG |
| Mini-XhoI-R | CCCGCTCGAGTCAATTTTAGAAATGATGCATCTTTCAAGCA |
| Mini-3F4R-F | ACTATTGCTCTCTTCATTTATTTGGC |
| Mini-3F4R-R | GCACATGAAAACGTGACTCCTG |
| Mini-3F5R-F | TATTGCTCTCTTCATTTATTTGGC |
| Mini-3F5R-R | GAAATGATGCATCTTTCAAGCA |
| qPCR-ACTB-F | GCACAGAGCCTCGCCTT |
| qPCR-ACTB-R | GTTGTCGACGACGAGCG |
| qPCR-CHD1L-F | CAGCGCTTCCATTGTCAGAA |
| qPCR-CHD1L-R | CTCCTTGTCGCCTGCATATG |
